# Seasonal Influenza Vaccination Coverage Rate of Target Groups in Selected Cities and Provinces in China by Season (2009/10 to 2011/12)

**DOI:** 10.1371/journal.pone.0073724

**Published:** 2013-09-09

**Authors:** Lei Zhou, Qiru Su, Zhen Xu, Ao Feng, Hui Jin, Shiyuan Wang, Zijian Feng

**Affiliations:** 1 Public Health Emergency Center, Chinese Center for Disease Control and Prevention, Beijing, China; 2 Key Laboratory of Surveillance and Early-warning on Infectious Disease, Division of Infectious Disease, Chinese Center for Disease Control and Prevention, Beijing, China; 3 School of Public Health, Southeast University, Nanjing, China; Centers for Disease Control and Prevention, United States of America

## Abstract

**Background:**

The objectives of the survey were to identify the level of influenza vaccination coverage in China in three influenza seasons 2009/10 to 2011/12, and to find out potential predictors for seasonal influenza vaccination.

**Methods:**

In September and October 2011, representative urban household telephone surveys were conducted in five provinces in China with a response rate of 6%. Four target groups were defined for analysis: 1) children ≤5 years old; 2) elderly persons aged ≥60 years old; 3) health care workers (persons working in the medical field) and 4) chronically ill persons.

**Results:**

The overall mean vaccination rate was 9.0%. Among the four target groups, the rate of vaccination of children aged ≤5 years old (mean = 26%) was highest and the rate of elderly people aged ≥60 years old (mean = 7.4%) was the lowest, while the rates of persons who suffer from a chronic illness (mean = 9.4%) and health care workers (9.5%) were similar. A subsidy for influenza vaccination, age group, health care workers, suffering from a chronic illness and living in Eastern China were independent significant predictors for influenza vaccination.

**Conclusions:**

The seasonal influenza vaccination coverage rates among urban populations in selected cities and provinces in China were far below previously reported rates in developed countries. Influenza vaccination coverage rates differed widely between different target groups and provinces in China. Subsidy policy might have a positive effect on influenza vaccination rate, but further cost-effectiveness studies, as well as the vaccination rate associated factors studies are still needed to inform strategies to increase coverage.

## Introduction

Influenza vaccination has been shown to be the most effective preventive measure to reduce influenza virus infection and its related morbidity and mortality [1]–[4]. Although the primary strategy for preventing influenza is getting vaccinated against influenza virus annually – and despite public awareness and efforts by policy makers, physicians and health care providers – influenza vaccination rates and vaccine manufacturing capacities need to be increased substantially to achieve the degree of baseline preparedness required in case of a surge in immunization uptake when an influenza pandemic occurs, both for the world and China.

Every year, the World Health Organization (WHO) recommends to vaccine manufacturers what the seasonal influenza vaccine composition for the Northern and Southern Hemispheres should be. The influenza vaccine used in China belongs to the Northern Hemisphere, and the recommended vaccine virus strains of the most recent three influenza seasons (2009/10, 2010/11 and 2011/12) for the Northern Hemisphere are available on the WHO website [5]–[7].

In China, seasonal influenza vaccination is not included in the national immunization program and is therefore not subsidized. Recipients must purchase influenza vaccine except for a few cities where local government subsidy programs have been introduced. The Beijing municipal government subsidized influenza vaccination for people ≥60 years old and school-age children from 2007 to 2008 and provided free vaccines to these two population groups since 2009 [8]. Although the effectiveness of the subsidy vaccination strategy needs to be evaluated, it may influence influenza vaccination rates in Beijing. Additionally, the 2009 pandemic caused by the influenza A (H1N1) virus alerted the public to the harmfulness of influenza infection, and that event may have stimulated seasonal influenza vaccination rates in subsequent years.

For the purposes of this study, we assumed that seasonal influenza vaccination rates in urban regions in China may have different spatial and temporal features. This study was conducted in order to examine the level of seasonal influenza vaccination coverage from the 2009/10 influenza season to the 2011/12 one among different target groups in urban regions in China, and to explore potential predictors for seasonal influenza vaccination.

## Methods

### Design, Setting and Participants

The survey population were non-institutionalized persons of all ages in a private household living in urban areas of five provinces/municipalities in Mainland China. These five provinces/municipalities were selected according to geographic location and economic level as reported in the National Statistical Report [9] and divided into three categories by locations: 1) Eastern China including Beijing and Shandong; 2) Central China including Hunan and Henan and 3) Western China including Sichuan. Except for Beijing municipality, in each of the four provinces the capital city and a randomly selected prefecture were selected and all survey cities were assigned to one of three categories by urban residence population size [10]: 1) The Large City Category (>6 million urban people): Beijing, Chengdu (Sichuan) and Zhengzhou (Henan); 2) The Medium City Category (3∼6 million urban people) included two provincial capital cities: Changsha (Hunan) and Jinan (Shangdong); and 3) The Small City Category (<3 million urban people) included four common prefectures: Chenzhou (Hunan), Mianyang (Sichuan), Weifang (Shandong) and Xinxiang (Henan).

The study was a telephone survey conducted from September 15 through October 15, 2011. The sample households of each study city were chosen by random-digit dialling by using a computer-generated list of possible telephone numbers. Phone call dialling would not be completed until the number of participant households reached at least 1,000 in each province. The person with age ≥18 years old who initially answered the telephone was eligible to be interviewed and provide information on all family members, which required the person to have sufficient knowledge of all family members. If the person who answered the telephone did not meet the above criteria, they were asked if another person who met the eligibility criteria were available. If no eligible person was present in the household at the time of the call, an appointment was made and at most two attempts were made on the same day to conduct the interview. Data on all household members was collected if the proxy responder met our inclusion criteria regardless whether there was a target group person or not. After oral informed consent was obtained from the informant, a standardized, pretested questionnaire was used to collect data on each member in the household, including age, sex, working place, underlying medical conditions and history of seasonal influenza vaccination. In the influenza season 2009/10, there were two types of influenza vaccines in use: the seasonal and pandemic vaccines. Because the pandemic vaccine was free for vaccination, the participants were asked to only provide seasonal vaccination uptake status according to the price of vaccine.

China's guidelines for seasonal influenza vaccination were adapted from the recommendations of the United States Advisory Committee on Immunization Practices (ACIP) [11] and had been issued annually by the Chinese Center for Disease Control and Prevention (China CDC) since 2007 [12]. Four categories of target groups for analyses based upon seasonal influenza vaccination recommendations of China CDC were determined as follows:

1. Children ≤5 years old;
2. Persons ≥60 years old;
3. Chronic illness: Individuals who suffered from a chronic illness, which was defined as chronic pulmonary (including asthma) or cardiovascular (except isolated hypertension), renal, hepatic, neurological, hematologic, or metabolic disorders (including diabetes mellitus).
4. Health care workers: Individuals who were working in the medical field, such as doctors, nurses, clinical laboratory staff, administrative staff, or other staff in medical facilities.

Participants were classified by age for each of the three influenza seasons separately. During the interview, we asked the interviewee the age of each person in the household and recorded it. We asked additional questions to confirm the age in previous influenza seasons if it was possible the person had changed age groups. Participants were included in more than one group if applicable; for example, a 61 year old physician would be in both persons ≥60 and working in the medical field.

### Analysis

Only Beijing subsidized seasonal influenza vaccine [8], therefore data were categorized and analyzed by residence in Beijing; all including Beijing, excluding Beijing and only Beijing. Means and standard errors were calculated for continuous variables, and were compared between different categories using the Wilcoxon rank sum test. For categorical variables, frequencies for different categories were compared using Fisher's exact test. For multivariate logistic regressions, we did the analysis three ways, all including Beijing, excluding Beijing and only Beijing. Since all variables, including age group, sex, health care workers, suffering from a chronic illness, location and city category, and subsidy policy, could be potential predictors for vaccination, we included all variables for the full model regression analyses using all data including Beijing. Variables with more than two categories were viewed as a dummy variable in full model analyses, and the 15–59 age group, Small City and Western China were used as reference categories, respectively. Adjusted OR and 95% CIs were calculated for each predictor. All tests of statistical significance were 2-sided with a significance level of α = 0.05. Data were analyzed with SAS 9.1 (SAS Institute Inc, Cary, NC, USA, SN: 0050076001).

### Ethics statement

The study was reviewed and approved by the Chinese Center for Disease Control and Prevention Institute Review Board which is registered with the Office for Human Research Protections (active no. in 2012 was IRB 00005183) and has a US Federal Wide Assurance (FWA 00002896). As a study on medical record data with no patients contact and no collection of personal data, the Chinese Center for Disease Control and Prevention Institute Review Board waived the need for written informed consent from the participants.

## Results

### Study participants

**Figure 1:**
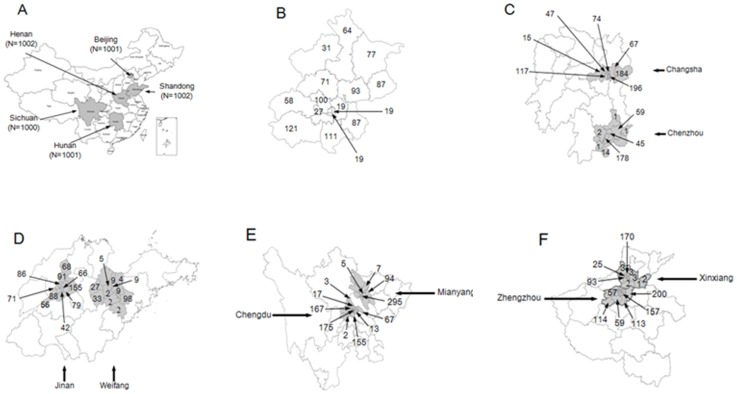
Geography distribution of 5006 telephone numbers. Figure 1 describes the geography distribution of 5006 telephone numbers by province and city. A was distribution in China. B was distribution in Beijing Municipal. C was distribution in Hunan Province. D was distribution in Shandong Province. E was distribution in Sichuan Province. F was distribution in Henan Province.

Among 169,847 phone numbers dialled, 87,483 (52%) were actual connections at a private household. Of these, 5,006 (6%) agreed to participant in the study, including Beijing (1001), Hunan (1001), Shandong (1002), Sichuan (1000) and Henan (1002). The geographic distribution of urban participant households is shown in Figure 1. The remaining 82,477 (94%) households were discarded because the person who answered the phone refused to participate. In the 5,006 (6%) participating urban households, information was collected on 23,220 persons. The number of participants of the four influenza vaccination target groups was respectively 1,523 children ≤5 years old, 5,414 people ≥60 years old, 576 health care workers and 1,276 individuals suffering from a chronic illness (Table 1). No significant differences by sex, location or city size categories were seen.

**Table 1:** Overview of study participants.

	Total (N = 23220)	Eastern China (n = 9427)	Central China (n = 9320)	Western China (n = 4473)	Large City (n = 10564)	Medium City (n = 7097)	Small City (n = 5559)	Beijing (n = 4555)
Age group (years) (%)								
≤5	1523 (6.6)	646 (6.9)	648 (7.0)	229 (5.1)	675 (6.4)	458 (6.5)	390 (7.0)	272 (6.0)
6–14	2147 (9.2)	858 (9.1)	897 (9.6)	392 (8.8)	838 (7.9)	771 (10.9)	538 (9.7)	304 (6.7)
15–59	14136 (60.9)	5717 (60.6)	5657 (60.7)	2762 (61.7)	6611 (62.6)	4155 (58.5)	3370 (60.6)	2887 (63.4)
≥60	5414 (23.3)	2206 (23.4)	2118 (22.7)	1090 (24.4)	2440 (23.1)	1713 (24.1)	1261 (22.7)	1092 (24.0)
Male (%)	11566 (49.8)	4656 (49.4)	4658 (50.0)	2252 (50.3)	5278 (50.0)	3491 (49.2)	2797 (50.3)	2256 (49.5)
Health care workers (%)	576 (2.5)	203 (2.2)	261 (2.8)	112 (2.5)	254 (2.4)	155 (2.2)	167 (3.0)	104 (2.3)
Suffering from a chronic illness (%)	1276 (5.5)	556 (5.9)	475 (5.1)	245 (5.5)	618 (5.9)	370 (5.2)	288 (5.2)	324 (7.1)

### Vaccination rates by target groups

The overall mean vaccination rate of the 23,220 urban participants surveyed was 9.0% (9.4% in 2009/10 season, 11.3% in 2010/11 season and 6.4% in 2011/12 season) (Table 2). Among the four target groups, the vaccination rate of children≤5 years (mean = 26%) was the highest while that of people aged ≥60 years (mean = 7.4%) was the lowest. The rate of persons who suffer from a chronic illness (mean = 9.4%) and health care workers (mean = 9.5%) were similar. If we excluded the Beijing data, the vaccination rate and order of four target groups were similar to the results using data including Beijing, except the vaccination rate of people ≥60 years old falls to a mean of 4.1% (Table S1).

**Table 2:** Number of seasonal influenza vaccinated and coverage rates in three influenza seasons (2009/10, 2010/11 and 2011/12) in five selected provinces in China.

No. of vaccinated (VCR, %)	Mean VCR (%)	2009/10 season	*p* value	2010/11 season	*p* value	2011/12 season	*p* value
Overall	9.0	2192 (9.4)		2613 (11.3)		1495 (6.4)	
Age group (years) : ≤5	26.4	333 (21.9)	**<0.001**	481 (31.6)	**<0.001**	390 (25.6)	**<0.001**
6–14	29.5	680 (31.7)		772 (36.0)		444 (20.7)	
15–59	4.7	718 (5.1)		844 (6.0)		427 (3.0)	
≥60	7.4	461 (8.5)		516 (9.5)		234 (4.3)	
Sex: Male	9.2	1098 (9.5)	0.782	1315 (11.4)	0.576	767 (6.6)	0.232
Female	8.9	1094 (9.4)		1298 (11.1)		728 (6.2)	
Health care workers	9.5	62 (10.8)	0.271	70 (12.2)	0.489	32 (5.6)	0.382
Suffering from a chronic illness	9.4	141 (11.1)	**0.043**	152 (11.9)	0.444	67 (5.3)	0.075
By location: Eastern China	11.8	1049 (11.1)	**<0.001**	796 (17.5)	**<0.001**	630 (6.7)	**0.012**
Central China	8.5	792 (8.5)		955 (10.2)		621 (6.7)	
Western China	7.7	351 (7.8)		440 (9.8)		244 (5.5)	
By city size: Large City	13.6	688 (15.1)	**<0.001**	796 (17.5)	**<0.001**	380 (8.3)	**0.008**
Medium City	7.7	562 (7.9)		660 (9.3)		424 (6.0)	
Small City	8.2	467 (8.4)		562 (10.1)		333 (6.0)	

VCR: vaccination coverage rate.

### Vaccination rates by categories

The vaccination rate of Eastern China was the highest (mean = 12%) among three location categories. The vaccination rate of Large City (mean = 14%) was higher than those of Medium (mean = 7.7%) and Small City (mean = 8.2%) (Table 2). Similar vaccination rates around 8% were found between Central and Western China, and between Medium and Small City. But if we excluded the Beijing data, the mean vaccination rate of Eastern China and Large City was dropped to 7.1% and 7.9% (Table S1).

### Vaccination rates in Beijing

The mean vaccination rate of Beijing was 16% and rate of primary and junior school-age children (age 6–14 years old) was the highest (mean = 39%). Among the target groups, the mean vaccination rates of elderly (aged≥60 years old), health care workers and persons suffering from a chronic illness in Beijing were 21%, 13% and 21%, respectively, which were higher than the mean rate in the other four selected provinces (4.1%, 8.8% and 7.5%) (Table S1).

### Predictors for vaccination

Univariate analyses using data of provinces including Beijing indicated age group, suffering from a chronic illness, location and city size were statistically significant predictors (Table 2). Influenza vaccination subsidy policy, age group, health care workers, suffering from a chronic illness and living in Eastern China in the full model multivariate analyses using data including Beijing were statistically significance with α<0.05 (Table 3). Excluding Beijing, age≥60 years old was not a significant predictor (Table S2). For Beijing data, only age group and suffering from a chronic illness in the full model were found to be statistically significant (p<0.05) (Table S2).

**Table 3:** Multivariate analyses results of predictors for seasonal influenza vaccination in three seasons (2009/10–2011/12)*.

Predictors	2009/10 season		2010/11 season		2011/12 season		Any one of seasons	
	Adjusted OR	95% C.I.	Adjusted OR	95% C.I.	Adjusted OR	95% C.I.	Adjusted OR	95% C.I.
Influenza vaccination subsidy policy	3.112	2.536-3.82	3.137	2.589–3.801	2.478	1.948–3.151	2.971	2.493–3.540
≤5 yrs†	5.855	5.058–6.777	8.246	7.224–9.412	11.897	10.209–13.864	9.441	8.356–10.666
6–14 yrs†	10.200	9.027–11.526	10.438	9.29–11.728	9.154	7.919–10.582	10.762	9.657–11.994
≥60 yrs†	1.656	1.458–1.881	1.597	1.417–1.801	1.400	1.182–1.658	1.416	1.27–1.579
Health care workers	2.209	1.673–2.916	2.169	1.666–2.823	1.898	1.311–2.749	2.163	1.705–2.745
Suffering from a chronic illness	1.612	1.324–1.963	1.513	1.252–1.830	1.406	1.072–1.843	1.596	1.343–1.896
Male	0.991	0.903–1.088	0.996	0.912–1.087	1.022	0.916–1.142	1.007	0.929–1.091
Large City‡	0.931	0.807–1.073	0.962	0.844–1.097	0.967	0.821–1.14	0.816	0.725–0.919
Medium City‡	0.984	0.848–1.142	0.984	0.856–1.131	1.11	0.936–1.315	0.886	0.782–1.004
Eastern China ^§^	0.787	0.646–0.959	0.720	0.599–0.865	0.700	0.556–0.881	0.698	0.592–0.822
Central China ^§^	1.014	0.876–1.173	0.964	0.843–1.103	1.087	0.917–1.288	0.910	0.807–1.028

C.I.: Confidence Interval.

OR: Odds ratio.

* All variables, including age group, sex, health care workers, suffering from a chronic illness, location and city category, and subsidy policy, were included for the full model regression analyses in the multivariable analysis.

† Reference category: people 15–59 years old.

‡ Reference category: Small City.

## Discussion

The overall coverage rates of the study population (mean = 9.0%) was lower than that in the Republic of Korea (34%) [13]. When comparing with the coverage rates in European countries, the rate in our study was around one third of the coverage rates reported in higher per capita gross national income (range: US$ 19,276.10–24,486.70) European countries [14]–[16], and was similar with the eastern European countries which were similar to China in social and economic situation (e.g. 9.5% in Poland in season 2007/08) [17]. Among the four target groups, although the vaccination rates of children ≤5 years old (mean = 26%) was higher than in the other target groups, it was still lower than that in the United States (around 40% in seasons from 2006/07 to 2008/09) [18]–[20]. When comparing with the European countries, the rates of children ≤5 years old in our study was higher than that in higher per capita gross national income European countries, e.g. 5–17% in France in 2003 [21], 3–6% in Israel in season 2003/04 [22], 7% in Spain in 2005 [23], around 5% in Ireland and around 10% in England in season 2006/07–2007/08 [17], and much higher than Poland (2% during 2004–2008) [24]. In addition to the children's target group, the rates of persons≥60 years , health care workers and suffering from a chronic illness (around 11%) were far below that in developed countries: rates of these three target groups in the United States, Canada, New Zealand, and European countries e.g. England, Germany, France, Hungary and Spain are between 37–82% [14], [15], [17], [25]–[28], 16–68% [15], [16], [17], [25], [28]–[32] and 24–84% [15], [16], [17], [25], [33]–[39], respectively. As the city with influenza vaccination subsidy policy among school-age children and elderly, Beijing attained seasonal influenza vaccination rates of target groups (12–39%) that were relatively comparable with developed countries.

Predictor analysis results highlighted the complex factors influencing seasonal influenza vaccination in China. As noted earlier, the Beijing municipal government has had a policy of subsidized seasonal influenza vaccination since 2007 [8] and the subsidy policy was one of the significant predictors, indicating that subsidy policy in the target populations, i.e. children and elderly, might have had a positive effect on vaccination rates. A previous study conducted by a vaccine company in 10 countries also indicated that the lack of an influenza vaccination reimbursement strategy might be associated with lower vaccination rates, and the rates of countries without free influenza vaccine were usually lower than comparable countries with free influenza vaccine by about 10% [40]. A positive correlation of vaccination coverage rate with vouchers for free or reimbursed vaccination was also explored in a previous study in 16 European countries [41]. But it cannot be concluded directly that the demands of seasonal influenza vaccination of Chinese people would increase with lower vaccine price. In this study, excluding Beijing data, the vaccination rate of Eastern China was lower than those of Central and Western China. The factor of being in Eastern China is negatively correlated with vaccination uptake, indicating that the economic factor (as indicated by the people's province of residence) might not be as important as we expected for people's decision to receive influenza vaccination. Further study is needed to evaluate the impact and cost-effectiveness of a subsidy policy on seasonal influenza vaccine rates.

Besides economic factors, influenza vaccines acceptance might be influenced by the knowledge or attitude of vaccinees. Children≤5 years old and primary and junior school-age children aged 6–14 years old composed a special group which obtained much more attention from parents and society in China than outside attention for persons ≥60 years old and persons suffering from a chronic illness, which might explain the relatively high coverage rates among the children groups. The health care workers should have knowledge of seasonal influenza vaccination, but their vaccination rate was lower than the other target groups. Further studies about these factors and how they influenced influenza vaccination in China are needed to inform strategies to increase vaccination coverage among health care workers.

Results of this study should be interpreted with caution because of its several limitations. First, the response rate was only 6%, which might be the fundamental flaw of our study. The participants might be more positive and active concerning seasonal influenza vaccination than the non-responders. In addition, the percentage of household with telephones in the urban areas we studied (Beijing 70%, Shandong 56%, Henan 77%, Hunan 53% and Sichuan 53%) [9] indicated that almost half of the urban households still had no landlines, which also introduced participant bias. To validate the representation of participants, we collected the location of participants. The geographic distribution, as shown in Figure 1, illustrated that the responders were distributed equally throughout the study area. This potential participant selection bias might over-estimate the true vaccination rate although it seemed far below than those in the other countries. Second, the survey was conducted from mid-September to mid-October. While the annual seasonal influenza vaccination campaign in China usually started from September to March in the next year. According to the number of purchased vaccine, most of people were vaccinated at the first three months of the vaccination campaign duration (authors' unpublished data). Our study duration only covered one third of the 2011/12 influenza season, which might under-estimate the true vaccination coverage rates.

We were not able to confirm the self-reported vaccination uptake or chronic condition status. Because influenza vaccination was not included in the national immunization program, there was no medical record of influenza vaccination for verification in China. Although the interviewer used specific event information to assist responders to recall information, the recall bias could be introduced on the self-report seasonal influenza vaccination history. Meanwhile, although interviewer used the price of vaccine as one indicator to clarify the pandemic 2009 vaccine and seasonal influenza vaccine, there still had possibility to introduce recall bias because both pandemic 2009 vaccine and seasonal influenza vaccine were provided during influenza season 2009/10. Proxy respondent bias was another limitation as the responder provided information for other household members. Nevertheless, findings of previous studies supported the assertion that self-reported data of vaccination are reliable when compared with medical records [42], [43], and can be used to estimate reliably influenza vaccination rates [25], [44]–[47] . To increase the reliability of self-report data, a pilot study could be implemented to test the data. Furthermore, a comparable face-to-face survey can be carried out in the future to validate the reliability of self-report data.

Incomplete telephone registration records might also pose potential barriers to implementing telephone surveys in China. Because we had no detailed personal contact information, such as home address or working address through the telephone registration records, we could not trace the non-responders and then compare the characteristics of responders and non-responders. Likewise, we cannot compare the characteristics of the households with and without telephone because we lacked detailed information. However, this study nonetheless provides experience on implementing telephone surveys for public health strategies in China.

Our results illustrated the low seasonal influenza vaccination coverage among people living in urban China. It was highly relevant to the Chinese government's policy to increase the use of seasonal influenza vaccination, especially in these targeted populations. These findings also could have implications for specific influenza vaccination strategies targeting different target populations, and different urban geographic areas in China. Such insights could eventually help improve overall seasonal influenza vaccine coverage levels and reduce influenza disease burden in China. The data from Beijing suggested that subsidizing seasonal influenza vaccination policy could increase coverage in other provinces/municipalities. Its potential impact warranted more in-depth evaluation. Establishing a medical record system based on the immunization service points in China should improve immunization program management and monitoring. Improving the telephone registration system would also afford opportunities to improve immunization program management and monitoring.

## Conclusions

The seasonal influenza vaccination coverage rates among urban populations in selected cities and provinces in China were far below previously reported rates in developed countries. Influenza vaccination coverage rates differed between different target groups and varied among different regions in China. Subsidy policy might have a positive effect on influenza vaccination rate, but further evaluation and cost-effectiveness studies, as well as the vaccination rate associated factors studies are still needed to inform strategies to increase coverage.

## Supporting Information

Table S1 **Number of seasonal influenza vaccinated and coverage rates in three influenza seasons (2009/10, 2010/11 and 2011/12) in Beijing only and Beijing excluding provinces.** Table S1 showed the influenza vaccination results in Beijing and provinces excluding Beijing. Provinces excluding Beijing included Hunan, Henan, Sichuan and Shandong provinces.
(DOCX)

Table S2 **Multivariate analyses results of predictors for seasonal influenza vaccination in three seasons (2009/10**–**2011/12).** Table S2 showed the results of two multivariable analysis using data excluding Beijing and only Beijing. All variables, including age group, sex, health care workers, suffering from a chronic illness, location and city category, were included for the full model regression analyses when analyzed using data excluding Beijing. Only age group, sex, work in a medical field and suffering from a chronic illness were included for the model when amazed using only Beijing data. Provinces excluding Beijing included Hunan, Henan, Sichuan and Shandong provinces.^§^ Reference category: Western China.
(DOCX)
