# Supplementary material for: Seasonal Influenza Vaccination Coverage Rate of Target Groups in Selected Cities and Provinces in China by Season (2009/10 to 2011/12)
Source: PLoS One. 2013 Sep 9;8(9):e73724. doi: 10.1371/journal.pone.0073724 (PMC3767785; doi:10.1371/journal.pone.0073724)
Supplement: Table S1 — Number of seasonal influenza vaccinated and coverage rates in three influenza seasons (2009/10, 2010/11 and 2011/12) in Beijing only and Beijing excluding provinces. Table S1 showed the influenza vaccination results in Beijing and provinces excluding Beijing. Provinces excluding Beijing included Hunan, Henan, Sichuan and Shandong provinces. (DOCX) [file pone.0073724.s001.docx]

Table S1. Number of seasonal influenza vaccinated and coverage rates in three influenza seasons (2009/10, 2010/11 and 2011/12) in Beijing only and Beijing excluding provinces

| No. of vaccinated (VCR, %) | | Mean VCR (%) | 2009/10 season | *p* value | 2010/11 season | *p* value | 2011/12 season | *p* value |
| --- | --- | --- | --- | --- | --- | --- | --- | --- |
| Overall | Excluding Beijing | 7.9 | 1504(8.1) |  | 1817(9.7) |  | 1115(6.0) |  |
|  | Beijing | 16.0 | 688 (17.8) |  | 796 (21.2) |  | 380 (9.1) |  |
| Age group (years) : ≤5 | Excluding Beijing | 27.8 | 288 (23.0) | **<0.001** | 410 (32.8) | **<0.001** | 347 (27.7) | **<0.001** |
|  | Beijing | 19.5 | 45 (16.5) | **<0.001** | 71 (26.1) | **<0.001** | 43 (15.8) | **<0.001** |
| 6-14 | Excluding Beijing | 27.9 | 552 (30.0) |  | 627 (34.0) |  | 364 (19.8) |  |
|  | Beijing | 38.7 | 128 (42.1) |  | 145 (47.7) |  | 80 (26.3) |  |
| 15-59 | Excluding Beijing | 3.9 | 467 (4.2) |  | 564 (5.0) |  | 290 (2.6) |  |
|  | Beijing | 7.7 | 251 (8.7) |  | 280 (9.7) |  | 137 (4.8) |  |
| ≥60 | Excluding Beijing | 4.1 | 197 (4.6) |  | 216 (5.0) |  | 114 (2.6) |  |
|  | Beijing | 20.9 | 264 (24.2) |  | 300 (27.5) |  | 120 (11.0) |  |
| Sex: Male | Excluding Beijing | 7.8 | 764 (8.2) | 0.458 | 893 (9.5) | 0.382 | 537 (5.7) | 0.177 |
|  | Beijing | 13.5 | 334 (14.8) | 0.576 | 391 (17.3) | 0.800 | 189 (8.4) | 0.932 |
| Female | Excluding Beijing | 8.0 | 740 (7.9) |  | 924 (9.9) |  | 578 (6.2) |  |
|  | Beijing | 13.8 | 354 (15.4) |  | 405 (17.6) |  | 191 (8.3) |  |
| Health care workers | Excluding Beijing | 8.8 | 50 (10.6) | **0.040** | 1767 (9.7) | 0.524 | 1090 (6.0) | 0.530 |
|  | Beijing | 12.5 | 12 (11.5) | 0.304 | 20 (19.2) | 0.633 | 7 (6.7) | 0.548 |
| Suffering from a chronic illness | Excluding Beijing | 7.5 | 63 (6.6) | 0.094 | 1753 (9.9) | **0.001** | 1086 (6.1) | **<0.001** |
|  | Beijing | 21.0 | 78 (24.1) | **<0.001** | 88 (27.2) | **<0.001** | 38 (11.7) | **0.022** |
| By location: Eastern China | Excluding Beijing | 7.1 | 361 (7.4) | 0.065 | 422 (8.7) | **0.010** | 250 (5.1) | **<0.001** |
| By city size: Large City | Excluding Beijing | 7.9 | 475 (7.9) | 0.533 | 595 (9.9) | 0.271 | 358 (6.0) | 0.997 |

VCR: vaccination coverage rate.

Excluding Beijing included four (Hunan, Henan, Sichuan and Shandong) provinces.
