# Supplementary material for: Seasonal Influenza Vaccination Coverage Rate of Target Groups in Selected Cities and Provinces in China by Season (2009/10 to 2011/12)
Source: PLoS One. 2013 Sep 9;8(9):e73724. doi: 10.1371/journal.pone.0073724 (PMC3767785; doi:10.1371/journal.pone.0073724)
Supplement: Table S2 — Multivariate analyses results of predictors for seasonal influenza vaccination in three seasons (2009/10–2011/12). Table S2 showed the results of two multivariable analysis using data excluding Beijing and only Beijing. All variables, including age group, sex, health care workers, suffering from a chronic illness, location and city category, were included for the full model regression analyses when analyzed using data excluding Beijing. Only age group, sex, work in a medical field and suffering from a chronic illness were included for the model when amazed using only Beijing data. Provinces excluding Beijing included Hunan, Henan, Sichuan and Shandong provinces.§ Reference category: Western China. (DOCX) [file pone.0073724.s002.docx]

Table S2. Multivariate analyses results of predictors for seasonal influenza vaccination in three seasons (2009/10-2011/12) ^*^

| Predictors |  | 2009/10 season | | 2010/11 season | | 2011/12 season | | Any one of seasons | |
| --- | --- | --- | --- | --- | --- | --- | --- | --- | --- |
|  |  | Adjusted OR | 95% C.I. | Adjusted OR | 95% C.I. | Adjusted OR | 95% C.I. | Adjusted OR | 95% C.I. |
| Influenza vaccination subsidy policy |  | 3.112 | 2.536-3.82 | 3.137 | 2.589-3.801 | 2.478 | 1.948-3.151 | 2.971 | 2.493-3.540 |
| ≤5 yrs^†^ | Excluding Beijing | 7.488 | 6.358-8.82 | 9.917 | 8.554-11.496 | 15.407 | 12.953-18.326 | 11.069 | 9.665-12.678 |
|  | Beijing | 2.111 | 1.494-2.982 | 3.389 | 2.515-4.568 | 3.837 | 2.652-5.552 | 4.155 | 3.157-5.469 |
| 6-14 yrs^†^ | Excluding Beijing | 10.647 | 9.267-12.232 | 10.428 | 9.151-11.883 | 9.787 | 8.287-11.559 | 10.662 | 9.46-12.018 |
|  | Beijing | 7.739 | 5.949-10.068 | 8.773 | 6.777-11.356 | 7.327 | 5.377-9.985 | 9.435 | 7.319-12.163 |
| ≥60 yrs^†^ | Excluding Beijing | 1.051 | 0.88-1.256 | 0.967 | 0.818-1.142 | 1.017 | 0.811-1.276 | 0.911 | 0.788-1.054 |
|  | Beijing | 3.143 | 2.581-3.827 | 3.325 | 2.754-4.014 | 2.322 | 1.778-3.033 | 3.018 | 2.524-3.610 |
| Health care workers | Excluding Beijing | 2.635 | 1.937-3.584 | 2.180 | 1.605-2.960 | 2.013 | 1.324-3.060 | 2.218 | 1.696-2.900 |
|  | Beijing | 0.947 | 0.509-1.765 | 1.583 | 0.948-2.643 | 1.127 | 0.514-2.47 | 1.398 | 0.848-2.306 |
| Suffering from a chronic illness | Excluding Beijing | 1.578 | 1.194-2.087 | 1.382 | 1.050-1.819 | 1.142 | 0.769-1.696 | 1.532 | 1.214-1.935 |
|  | Beijing | 1.442 | 1.079-1.926 | 1.46 | 1.104-1.929 | 1.45 | 0.988-2.126 | 1.465 | 1.119-1.919 |
| Male | Excluding Beijing | 0.994 | 0.889-1.111 | 0.989 | 0.891-1.097 | 1.026 | 0.902-1.167 | 1.018 | 0.926-1.118 |
|  | Beijing | 1.008 | 0.851-1.194 | 1.043 | 0.888-1.225 | 1.061 | 0.855-1.317 | 1.012 | 0.869-1.179 |
| Large City^‡^ | Excluding Beijing | 0.925 | 0.801-1.068 | 0.955 | 0.836-1.091 | 0.961 | 0.813-1.135 | 0.807 | 0.716-0.911 |
| Medium City^‡^ | Excluding Beijing | 1.008 | 0.867-1.171 | 1.006 | 0.874-1.159 | 1.137 | 0.957-1.351 | 0.901 | 0.794-1.023 |
| Eastern China ^§^ | Excluding Beijing | 0.746 | 0.611-0.911 | 0.680 | 0.565-0.819 | 0.661 | 0.523-0.834 | 0.664 | 0.562-0.784 |
| Central China ^§^ | Excluding Beijing | 0.983 | 0.848-1.140 | 0.935 | 0.815-1.072 | 1.056 | 0.889-1.255 | 0.886 | 0.783-1.002 |

C.I.: Confidence Interval.

OR: Odds ratio

Excluding Beijing included four (Hunan, Henan, Sichuan and Shandong) provinces.

^*^ We did two multivariable analysis using data excluding Beijing and only Beijing. All variables, including age group, sex, working in a medical field, suffering from a chronic illness, location and city category, were included for the full model regression analyses when analyzed using data excluding Beijing. Only age group, sex, work in a medical field and suffering from a chronic illness were included for the model when amazed using only Beijing data.

^†^ Reference category: people 15-59 years old.

^‡^ Reference category: Small City.

^§^ Reference category: Western China.
